# Adenovirus-mediated delivery of interferon-γ gene inhibits the growth of nasopharyngeal carcinoma

**DOI:** 10.1186/1479-5876-10-256

**Published:** 2012-12-28

**Authors:** Ran-yi Liu, Ying-hui Zhu, Ling Zhou, Peng Zhao, Hong-li Li, Lan-cai Zhu, Hong-yu Han, Huan-xin Lin, Liang Kang, Jiang-xue Wu, Wenlin Huang

**Affiliations:** 1 State Key Laboratory of Oncology in South China, Sun Yat-sen University Cancer Center, 651 Dong-feng Road East, Guangzhou, 510060, China; 2 Department of Medical Oncology, The First Affiliated Hospital, College of Medicine, Zhejiang University, Hangzhou, 310003, China; 3 Department of Gastrointestinal Oncology, Tianjin Medical University Cancer Institute and Hospital, Tianjin, 300060, China; 4 Department of Colorectal Surgery, the Sixth Affliated Hospital, Sun Yat-sen University, Guangzhou, 510655, China; 5 Institute of Microbiology, Chinese Academy of Science, Beijing, 100101, China; 6 Guangdong Provincial Key Laboratory of Tumor-targeted Drug, Guangzhou Doublle Bioproducts Co., Ltd., Guangzhou, 510663, China

**Keywords:** Gene therapy, Interferon-γ, Nasopharyngeal carcinoma, Adenoviral vector

## Abstract

**Background:**

Interferon-γ (IFN-γ) is regarded as a potent antitumor agent, but its clinical application is limited by its short half-life and significant side effects. In this paper, we tried to develop IFN-γ gene therapy by a replication defective adenovirus encoding the human IFN-γ (Ad-IFNγ), and evaluate the antitumoral effects of Ad-IFNγ on nasopharyngeal carcinoma (NPC) cell lines *in vitro* and in xenografts model.

**Methods:**

The mRNA levels of human IFN-γ in Ad-IFNγ-infected NPC cells were detected by reverse transcription-polymerase chain reaction (RT-PCR), and IFN-γ protein concentrations were measured by enzyme-linked immunosorbent assay (ELISA) in the culture supernatants of NPC cells and tumor tissues and bloods of nude mice treated with Ad-IFNγ. The effects of Ad-IFNγ on NPC cell proliferation was determined using MTT assay, cell cycle distribution was determined by flow cytometry analysis for DNA content, and cells apoptosis were analyzed by Annexin V-FITC/7-AAD binding assay and hoechst 33342/PI double staining. The anti-tumor effects and toxicity of Ad-IFNγ were evaluated in BALB/c nude mice carrying NPC xenografts.

**Results:**

The results demonstrated that Ad-IFNγ efficiently expressed human IFN-γ protein in NPC cell lines *in vitro* and *in vivo*. Ad-IFNγ infection resulted in antiproliferative effects on NPC cells by inducing G1 phase arrest and cell apoptosis. Intratumoral administration of Ad-IFNγ significantly inhibited the growth of CNE-2 and C666-1 cell xenografts in nude mice, while no significant toxicity was observed.

**Conclusions:**

These findings indicate IFN-γ gene therapy mediated by replication defective adenoviral vector is likely a promising approach in the treatment of nasopharyngeal carcinoma.

## Background

Nasopharyngeal carcinoma (NPC) is a rare tumor arising from the epithelium of the nasopharynx. However, it has a high incidence rate in South China and Southeast Asia [1]. Radiation therapy is the main strategy for local control of NPC [2], but the 5-year survival for stage IV NPC is only 30%. The poor survival is often associated with high incidences of local, regional and systemic recurrences. Although concurrent chemoradiotherapy is developed as a standard treatment approach for advanced NPC, the outcome isn’t still satisfactory [3]. Therefore, the development of multidisciplinary therapeutic approaches is crucial for improvement of survival in NPC patients.

Interferon-γ (IFN-γ), a multifunctional cytokine produced mainly by T helper cells, cytotoxic T cells and natural killer cells [4], exerts antiviral, antiproliferative, immunomodulatory and antiangiogenesis effects [5]. IFN-γ inhibits the growth of numerous tumors [6-10] and has been tried to use in the clinical management of tumors [11-14]. However, the clinical application of recombinant IFN-γ protein is hampered by its short half-life and significant side effects. IFN-γ Gene therapy can continuously produce and release therapeutic protein in local focus to overcome the obstacle of recombinant IFN-γ protein [9,10,15,16].

In this study, we tried to develop IFN-γ gene therapy by a replication defective adenovirus encoding the human IFN-γ (Ad-IFNγ) on nasopharyngeal carcinoma (NPC), our results showed Ad-IFNγ effectively expressed in NPC cells, significantly inhibited tumor cell proliferation and induced cell apoptosis *in vitro*, inhibited the growth of xenografts in nude mice.

## Methods

### Materials

Dulbecco’s Modified Eagle Medium (DMEM), fetal bovine serum, propidium iodide (PI), and TRIzol® reagent were from Invitrogen (Carlsbad, CA, USA). Reverse Transcription System and GoTaq® DNA Polymerase were from Promega (Beijing) Biotech Co., Ltd (Beijing, China). Annexin V-FITC/7-AAD apoptosis detection kit was from Beckman Coulter, Inc. (Marseille, France), while Double Stain Apoptosis Detection Kit (Hoechst 33342/PI) from GenScript USA Inc. (Piscataway, NJ, USA). Rabbit anti-human Ki-67 polyclonal antibody was from NeoMarkers For Lab Vision Corporation (Fremont, CA, USA), and Streptavidin-Horseradish Peroxidase kit from Beijing Zhongshan Golden Bridge Biotechnology Co. (Beijing, China). *In Situ* Cell Death Detection kit was purchased from Roche Applied Science (Mannheim, Germany). Human interferon-γ ELISA kit was purchased from Boster (Wuhan, China), recombinant human interferon-γ (rhIFN-γ) protein was from Shanghai Clonbiotech. Co., Ltd (Shanghai China). (3-(4,5-Dimethylthiazol-2-yl)-2,5-diphenyltetrazolium bromide (MTT) and all other reagents were of molecular biology grade and obtained from Sigma-Aldrich (Shanghai, China).

### Cell lines, recombinant adenoviruses and infection

Human nasopharyngeal carcinoma (NPC) cell lines CNE-1, CNE-2 and C666-1 were maintained in DMEM containing 10% fetal bovine serum (FBS) at a humidified atmosphere with 5% CO_2_ at 37°C. Replication defective adenoviruses encoding human interferon-γ (Ad-IFNγ) and β-galactosidase (Ad-LacZ), kindly provided by Guangzhou Doublle Bioproducts Co., Ltd., were stored at -80°C for use.

For adenovirus infection, NPC cells were seeded and cultured for 24 h, and then removed culture medium, washed with phosphate buffered saline (PBS) (pH 7.4), followed by infection with adenovirus in serum-free DMEM for 3 h. After removal of residual virus by PBS washing, cells were cultured in normal medium for indicated time before the analyses of IFN-γ expression, cell proliferation and apoptosis.

### Reverse transcription-polymerase chain reaction (PCR)

Cells were harvested and total RNAs were extracted using TRIzol® reagent according to the manufacture’s instruction. mRNAs were transcribed into cDNAs and PCR reactions were carried out using their specific primer pairs: IFN-γ’s sense, 5^′^- TTCAGCTCTGCATCGTTTTG-3^′^, antisense, 5^′^-TTACTGGGATGCTCTTCGAC-3^′^ (amplicon 473 bp); β-actin’s sense, 5^′^-CGTCTTCCCCTCCATCGTG-3^′^, antisense, 5^′^- TAGCACAGCCTGGATAGCAAC-3^′^ (amplicon 334 bp). Amplification was done with an initial cycle of 95°C for 4 min, followed by 30 cycles of 95°C for 30 s, 55°C for 50 s, 72°C for 30 s, with a final extension at 72°C for 10 min. PCR products were analyzed by ethidium bromide staining on 1.5% agarose gels.

### Enzyme-linked Immunosorbent Assay (ELISA)

NPC cells were seeded into 6-well plate at a density of 1×10^5^ cells/well for 24 hours and then infected with adenoviruses as described above. The culture supernatants were collected at different time points and IFN-γ concentration was determined by ELISA according to the manufacture’s instruction (Boster, Wuhan, China) (the sensitivity is 15.6 pg/mL).

### Cell proliferation analysis

The effect of Ad-IFNγ on NPC cell proliferation was determined using MTT assay as previously described [17]. Briefly, cells were seeded in 96-well plates at a density of 2000 cells/well for 24 hours and then infected with adenoviruses as described above, followed by incubation for 72 h. Viable cells were stained with MTT for 4 hours and followed by determination of OD_570 nm_ with a reference wavelength at 630 nm.

### Cell cycle distribution and apoptosis analysis

Cell cycle distribution was determined by flow cytometry analysis for DNA content. Briefly, cells were harvested by trypsinization and washed by PBS, and followed by fixed in cold 70% ethanol for 1 hour at 4º°C. Cell suspensions were washed twice in PBS, treated by ribonuclease and followed by PI staining. And then cells were performed flow cytometry analysis for DNA content and cell cycle distribution.

Cells apoptosis were analyzed by Annexin V-FITC/ 7-AAD binding assay and hoechst 33342/PI double staining. Both floating and adherent cells were harvested and washed by PBS, then followed by staining according to the manufacture’s instruction. Cells stained with Annexin V-FITC/7-AAD were analyzed by flow cytometry, while those stained with hoechst 33342/PI were analyzed by fluorescence microscopy.

### Animal model and experimental design

Female BALB/c nude mice (5-6 weeks old, 18-20 g) were obtained from Shanghai Slike Experimental Animals Co. Ltd. (License No. SCXK(hu)2003-0003), housed and fed under specific pathogen-free conditions (Certificate No. 26-2004C008) according to protocols approved by the Sun Yat-sen University Institutional Animal Care and Use Committee. All of the animal experiments were performed in accordance with Guidelines for the Welfare of Animals in Experimental Neoplasia. The pieces (about 1.5 mm in diameter) of CNE-2 or C666-1 tumors, which were maintained by serial subcutaneous transplantation in BALB/c nude mice, were subcutaneously transplanted into the flanks of mice to construct xenograft model.

To test the expression of Ad-IFNγ *in vivo*, mice were intratumorally injected with 2×10^9^ pfu in 100 μL of PBS when CNE-2 xenografts reached an approximate diameter of 7 mm. Tumor tissues and bloods were collected at different time. IFNγ concentration in the samples was measured by ELISA.

To assess antitumor effects of Ad-IFNγ *in vivo*, mice were randomly assigned into eight groups (7-8 mice per group) when xenografts reached 4-5 mm diameter. Mice were treated by weekly intratumoral injection of 100 μL of PBS, 2×10^9^ pfu (plaque-forming unit) of Ad-LacZ, 2×10^9^ pfu, 1×10^9^ pfu, 5×10^8^ pfu, or 1×10^8^ pfu of Ad-IFNγ (in 100 μL of PBS) per dose respectively; while mice in other two groups were treated by daily intratumoral injection of 100 μL of normal saline or 1×10^6^ IU/(kg body weight) of rhIFN-γ protein per dose respectively. The treatment was performed for three consecutive weeks, every injection was distributed equally into each half (8-12 mm of diameter) or quadrants (>12 mm of diameter) of tumors. Body weight and tumor size were measured every 4-5 days, and tumor xenografts were weighed at the end point of experiments.

### Histological analysis

Tumor tissue was fixed in buffered formalin and embedded in paraffin. Sections (5 μM thick) were mounted on Poly-L-Lysine treated slides, standard H&E staining was utilized for histopathological assay. Human Ki67 immunohistochemistry was performed for cell proliferation assay, while TUNEL labeling was done using a fluorescent *in situ* cell death detection kit for apoptosis analysis in tumor tissues.

### Statistical analysis

All experiments were repeated at least three times. The data were analyzed with One-way ANOVA or *t* test by using SPSS 10.0 for Windows software (SSPS Inc., Chicago, IL, USA). P < 0.05 was considered statistically significant.

## Results

### Ad-IFNγ efficiently expressed hIFNγ in NPC cells

**Figure 1:**
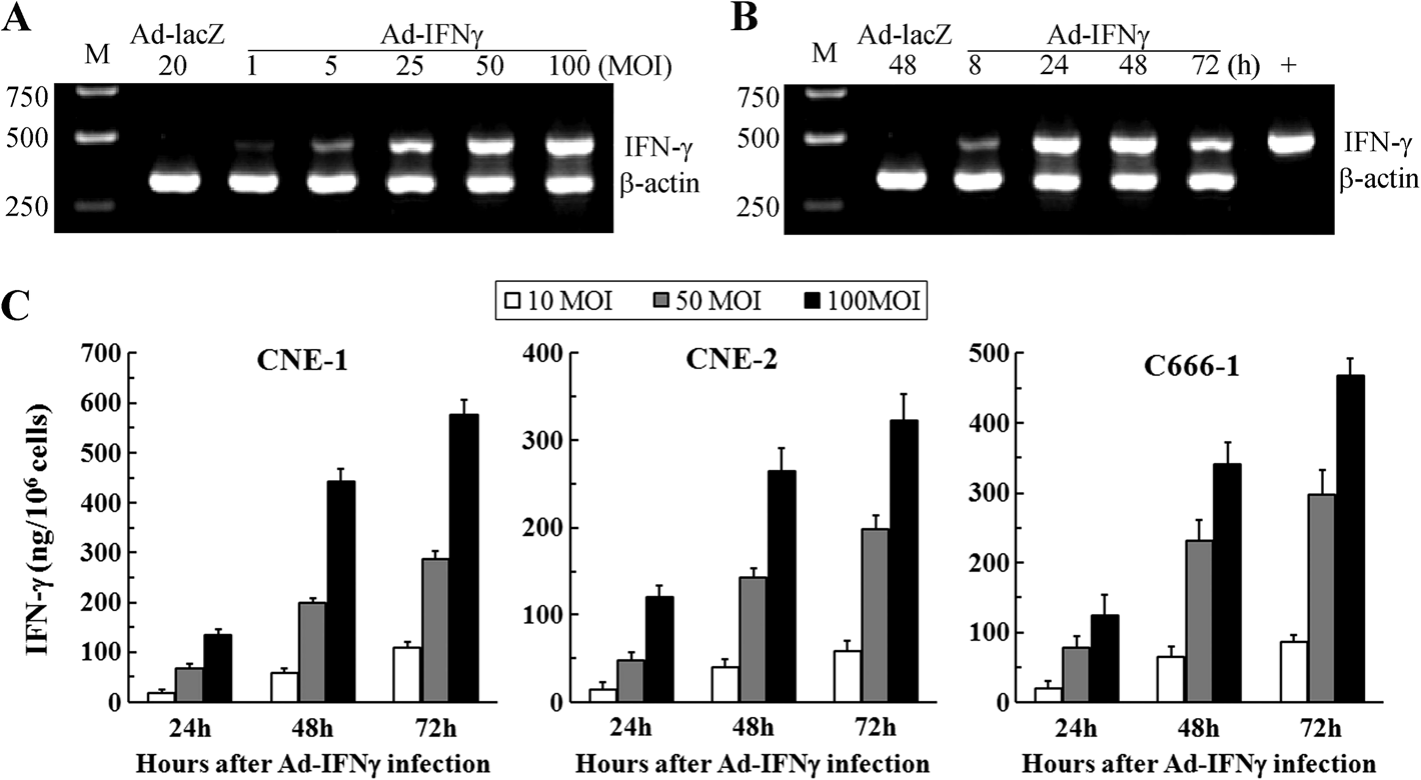
**Ad-IFNγ effectively expressed human IFN-γ in NPC cells. (A)** Dose-dependent IFN-γ expression at mRNA level. After CNE-2 cells were infected with Ad-IFNγ at different multiplicities of infection (MOIs) (20 MOIs of Ad-LacZ as negative control) for 24 hours, human IFNγ mRNA levels were detected by RT-PCR in CNE-2 cells (IFNγ 473 bp; human β-actin as internal control, 334 bp). **(B)** Time course of IFN-γ expression at mRNA level. IFNγ mRNA levels were detected by RT-PCR in CNE-2 cells infected with 50 MOIs of Ad-IFNγ for 8, 24, 48 or 72 hours, CNE-2 cells infected with 50 MOIs of Ad-LacZ for 48 hours were used as negative control. **(C)** IFN-γ expression at protein levels in NPC cells. CNE-1 (left), CNE-2 (middle) or C666-1 (right) cells were infected with Ad-IFNγ at indicated MOIs, the culture supernatants were then sampled at different time points, and IFN-γ concentrations in the supernatants were detected by ELISA (Boster, Wuhan, China). IFN-γ expression efficiency was displayed as ng/10^6^ cells.

To evaluate the capability of Ad-IFNγ expressed transgenic product in nasopharyngeal carcinoma (NPC) cells, we firstly investigated IFNγ mRNA levels in CNE-2 cells after Ad-IFNγ infection at different multiplicities of infection (MOIs) at different time. The results showed that Ad-IFNγ efficiently transcribed human IFNγ gene in CNE-2 cells at a dose-dependent manner (Figure 1A), hIFNγ mRNA was detected as early as 8 hours after Ad-IFNγ infection, the levels reached the top at 48 hours after infection, and then gradually decreased, while No IFNγ mRNA was detected in Ad-LacZ-infected cells (Figure 1B).

We investigated IFNγ expression at protein levels in CNE-1, CNE-2 or C666-1 infected by Ad-IFNγ, and found that IFNγ protein concentration in the supernatants of NPC cells continuously increased within 72 hours post-infection (Figure 1C). The differences from IFNγ mRNA which peaked at 48 hours may be due to the instability of the mRNA. No IFNγ proteins were found in the supernatants of Ad-LacZ-infected NPC cells (data not shown).

### Ad-IFNγ inhibited the proliferation of NPC cells *in vitro*

**Figure 2:**
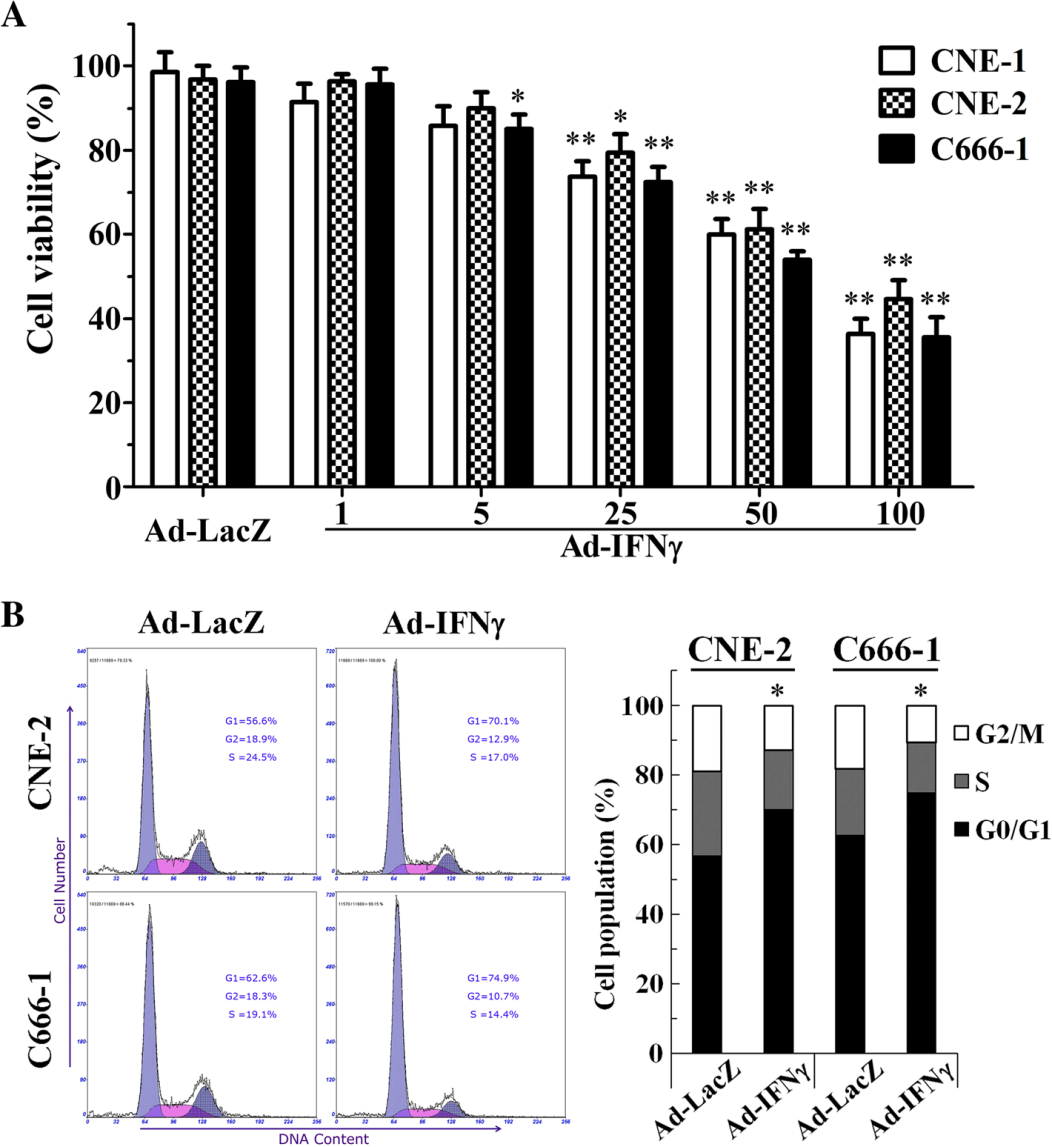
**Ad-IFNγ inhibited the proliferation of NPC cells by inducing G1 phase arrest *in vitro*. (A)** Cell viability assay. CNE-2 or C666-1 cells were infected with Ad-IFNγ at different MOIs for 72 h, followed by MTT assay for cell viability. **(B)** Cell cycle distribution. CNE-2 or C666-1 cells were infected with Ad-IFNγ at 50 MOIs for 48 h, followed by flow cytometry analysis for DNA content and cell cycle distribution as described in “Materials and methods”. ^*^ p < 0.05, ^**^ p < 0.01 while compared with corresponding cells infected with Ad-LacZ, N=3.

We evaluated the effects of Ad-IFNγ on the proliferation of nasopharyngeal carcinoma (NPC) cells by MTT assay after 72 h after Ad-IFNγ infection. The anti-proliferative effects of Ad-IFNγ on NPC cells were shown in Figure 2A. Ad-IFNγ inhibited the proliferation of CNE-1, CNE-2 and C666-1 NPC cell lines from 1 to 100 MOIs at a dose-dependent manner. No significant inhibition was observed on NPC cells after infection with 50 MOIs of Ad-LacZ.

To explore the mechanism involved in proliferation inhibition of Ad-IFNγ on NPC cells, the cell cycle profiles were analyzed by flow cytometry for DNA contents on NPC cells after infection with 100 MOIs of adenoviruses for 72 h. The results showed a significantly higher percentage at the G1 phase in the cells infected with Ad-IFNγ than those in Ad-LacZ infected cells (CNE-2: 70.8% ± 6.3% vs 56.1% ± 5.9%, p<0.05; C666-1: 75.8% ± 6.9% vs 62.0% ± 4.8%, p<0.05) (Figure 2B). These indicated that Ad-IFNγ possesses an effect of G1 phase arrest on NPC cells.

### Ad-IFNγ induced the apoptosis of NPC cells *in vitro*

**Figure 3:**
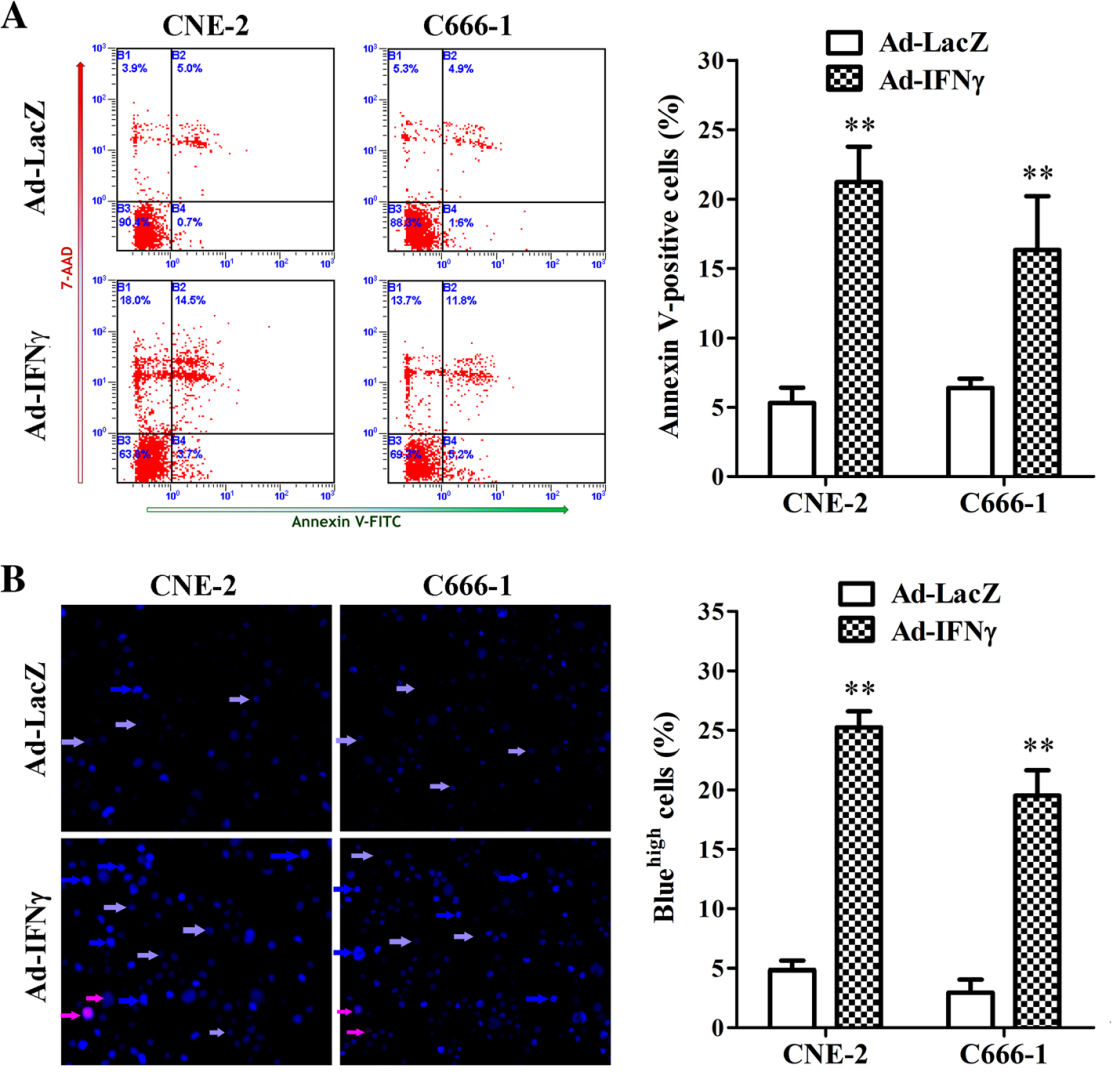
**Ad-IFNγ induced apoptosis in NPC cells.** Both floating and adherent NPC cells were harvested after infection with 50 MOI of Ad-IFNγ or Ad-LacZ for 72 hr, washed with ice-cold PBS and followed by apoptosis analysis. **(A)** Annexin V-FITC/7-AAD binding assay by flow cytometry. 5×10^5^ of collected cells were incubated in the dark for 15 minutes with 100 μL of 1×Binding Buffer containing 10 μL of Annexin V-FITC and 20 μL of 7-ADD Viability Dye (Beckman-Coulter, Inc. Marseille, France) on ice. Samples were diluted with 400 μL of 1×Binding Buffer and immediately analyzed by a Coulter Epics Altra flow cytometer (Beckman-Coulter). Left, Diagrams in a representative experiment. Annexin V^+^/7-AAD^−^ indicates early apoptotic cells, and Annexin V^+^/7-AAD^+^ indicates late apoptotic cells. Right, Statistical analysis of Annexin V-positive cells (apoptotic cells) generated from three independent experiments. **(B)** Hoechst 33342/PI double staining. Collected cells were adjusted to the density of 1×10^6^ cells/mL in PBS with 1% FBS and stained with 5 μM of Hoechst 33342 at 37°C for 10 min. And then cells were stained with 1 μM of PI for 10 min at room temperature after washing with PBS to remove Hoechst dye. The stained cells were mounted onto a polylysine-coated slide and examined under a fluorescent microscope. A total of 300~400 nuclei from 5~8 randomly chosen fields were examined. High blue fluorescent indicates apoptotic cells (bright blue arrow), low blue indicates live cells (azury arrow), while red represents dead cells (pink arrow). Apoptosis was expressed as a percentage of the total number of nuclei examined. Left, Representative pictures from one experiment. Right, Statistical analysis of apoptotic cells from three independent experiments. ** p<0.01, compared with Ad-LacZ-treated cells.

To examine the fate of Ad-IFNγ-infected NPC cell lines, an Annexin V/7-AAD binding assay and Hoechst 33342/PI double staining were performed after treatment with 100 MOIs of adenoviruses for 72 h. The results showed that there was an increased fraction of Annexin V^+^/7-AAD^−^ (early apoptosis) and Annexin V^+^/7-AAD^+^ (late apoptosis) in Ad-IFNγ-infected NPC cells than those of Ad-LacZ-infected cells (CNE-2, 21.2% vs 5.3%, p<0.01; C666-1, 16.3% vs 6.4%, p<0.01) (Figure 3A). In Hoechst 33342/PI double staining assay, there were higher percentages of bright blue cells (apoptosis) in NPC cells infected with Ad-IFNγ than those with Ad-LacZ (CNE-2, 25.2% vs 4.8%, p<0.01; C666-1, 19.5% vs 3.0%, p<0.01) (Figure 3B). These data suggested that Ad-IFNγ induced the apoptosis in NPC cells.

### Ad-IFNγ efficiently expressed human IFNγ in NPC xenografts in nude mice

**Figure 4:**
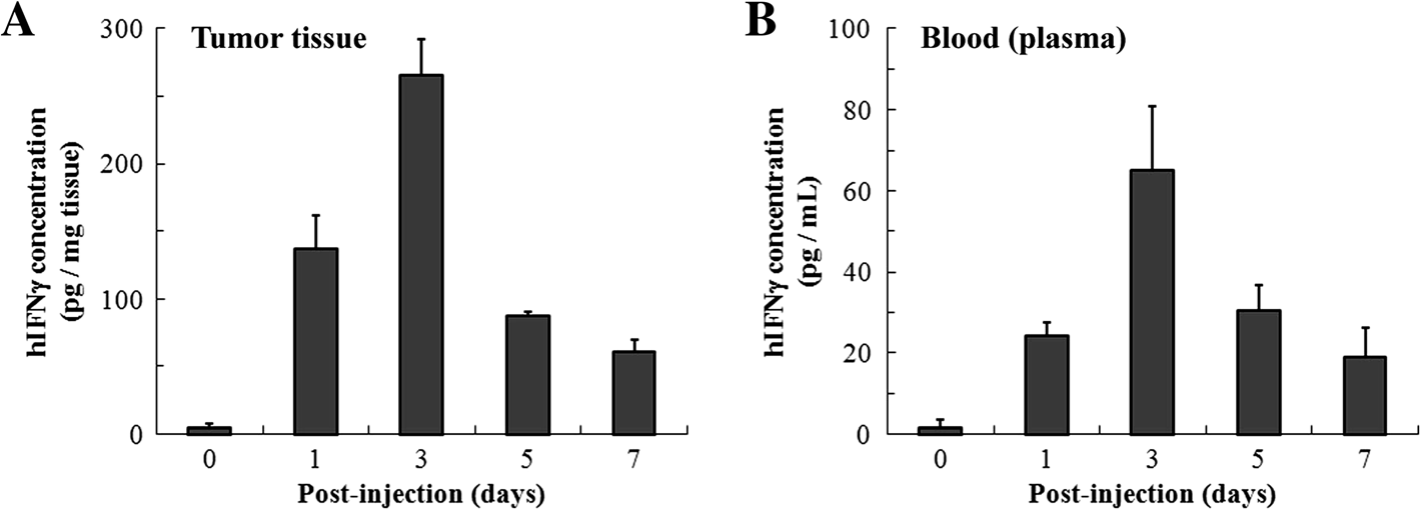
**Ad-IFNγ effectively expressed human IFN-γ in NPC xenografts in nude mice.** Mice were intratumorally injected with 2×10^9^ pfu of Ad-IFNγ, and then collected tumor tissues **(A)** and bloods **(B)** 1 day, 3, 5 and 7 days after Ad-IFNγ injection (mice before injection marked “day 0” as negative control, 3-4 mice per time point). Tumor tissues were made into homogenates in cold PBS, and then human IFNγ concentrations in the tissue homogenates and blood plasmas were measured by using human interferon-γ ELISA kit (Boster, Wuhan, China).

To evaluate the dynamic expression of AdIFNγ *in vivo*, we measured the levels of hIFNγ in tumor and blood samples collected from nude mice carrying CNE-2 NPC xenografts on days 1, 3, 5 and 7 after intratumoral injection with Ad-IFNγ. The results showed that Ad-IFNγ efficiently expressed hIFNγ in NPC xenografts, the concentration of hIFNγ in tumor tissue was (136.5±25.5) pg/(mg tissue) at 24 hours post-injection of AdIFNγ, reached the peak of (265.8±26.2) pg/(mg tissue) at 72 hours, and then decreased gradually to (61.1±8.3) pg/(mg tissue) at day 7 (Figure 4A). hIFNγ were also detected in blood. As same as in tumor tissue, the concentration of hIFNγ reached the peak of (64.9±15.9) pg/mL after 3 days post-injection, and then decreased gradually to (18.9±7.3) pg/mL at day 7.

### Ad-IFNγ inhibited the growth of human NPC xenografts

**Figure 5:**
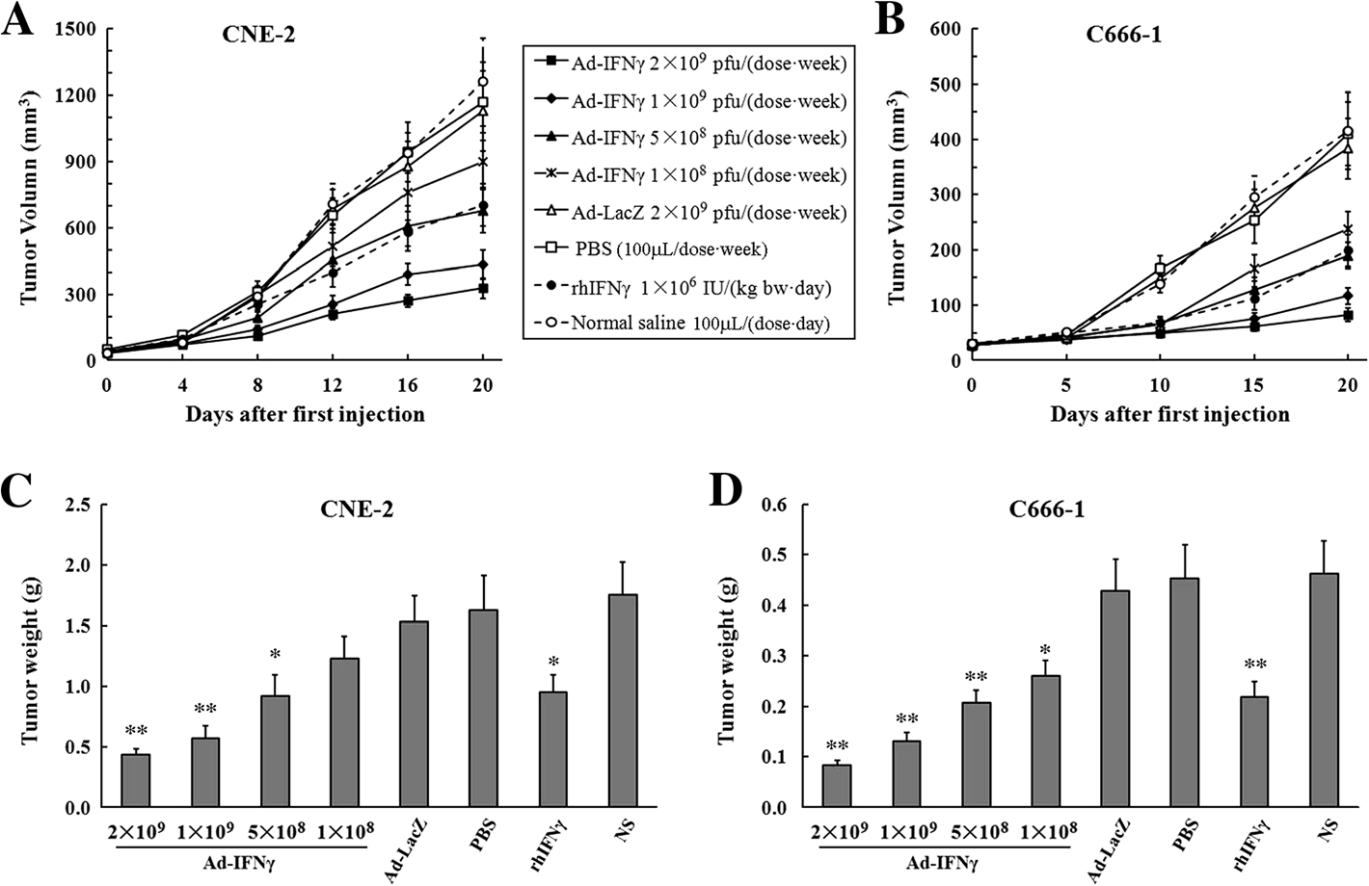
**Ad-IFNγ inhibited the growth of human NPC xenografts.** When CNE-2 or C666-1 xenografts reached 4-5 mm diameter, mice were randomly assigned (7~8 mice per group) and treated by weekly intratumoral injection of 100 μL of PBS, Ad-LacZ or Ad-IFNγ (in 100 μL of PBS) or by daily intratumoral injection of 100 μL of normal saline (NS) or rhIFN-γ protein (in 100 μL of NS), respectively. **(A, B)** Tumor growth curves. Tumor size was measured every 4 days for CNE-2 xenografts **(A)** or 5 days for C666-1 **(B)**, and the volume (V) was calculated according to the following formula: V = 0.52 × length × width^2^[18,19]. **(C, D)** Tumor weights. After 3 weeks of treatment, mice with CNE-2 **(C)** or C666-1 **(D)** xenografts were sacrificed and tumor tissues were resected followed by weighing. (* p < 0.05, ** p < 0.01 compared with corresponding media).

Administration of Ad-IFNγ resulted in significant growth suppression of NPC xenografts compared to the control groups. As shown in Figure 5A and 5B, tumor growth in the Ad-IFNγ-treated groups was significantly slower than that in Ad-LacZ (vector control) group or PBS (medium control) group. The inhibition effects showed a clear dose-dependent manner. At the end of experiment, the tumor weights in Ad-IFNγ groups were significantly lighter than those in Ad-LacZ or PBS group (Figure 5C and 5D), 1×10^8^, 5×10^8^, 1×10^9^ and 2×10^9^ pfu/dose of Ad-IFNγ treatment respectively produced 24.1%, 43.2%, 64.8%, and 73.5% of growth inhibition for CNE-2 xenografts (Figure 5C), 42.6%, 54.3%, 71.3% and 81.7% of growth inhibition for C666-1 xenografts (Figure 5D). However, 2×10^9^ pfu/dose of Ad-LacZ barely inhibited tumor growth. The efficacy of daily intratumoral injection of 1×10^6^ IU/(kg body weight)/day of rhIFNγ was likely similar with that of the administration of 5×10^8^ pfu/dose/week of Ad-IFNγ. There were no significant differences on body weights between Ad-IFNγ-treated and Ad-LacZ or PBS-treated mice (P>0.05, data not shown), which indicated low general toxicity on Ad-IFNγ treatment.

**Figure 6:**
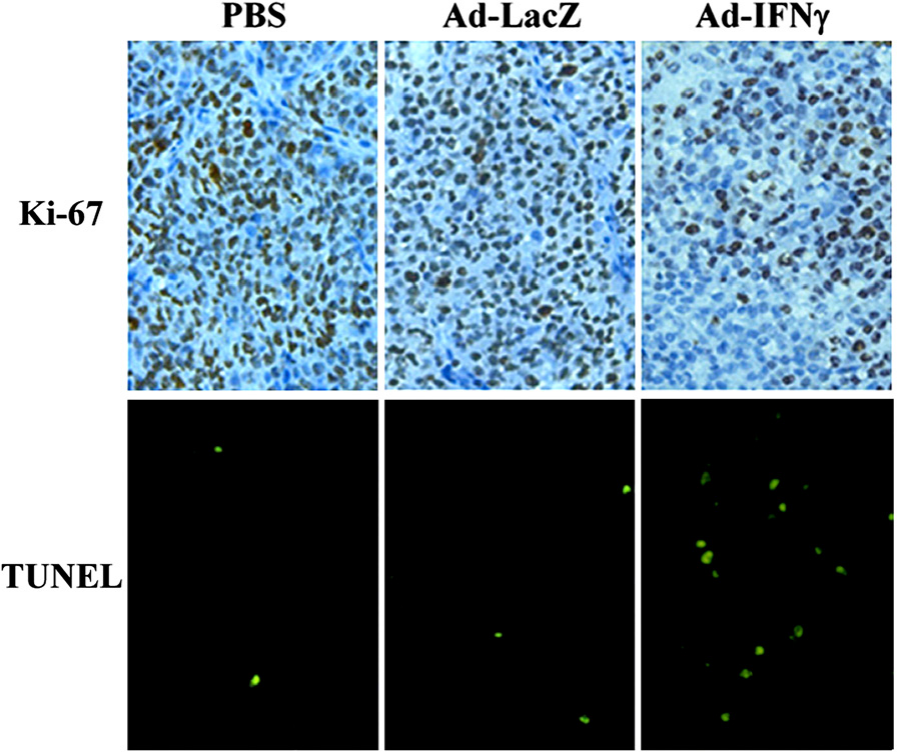
**Histological analysis in C666-1 xenografts.** C666-1 xenografts were resected at the end point of experiments, fixed and embedded in paraffin. Sections (5 μM thick) were mounted on Poly-L-Lysine treated slides. **Upper**, Ki67 expression assay. Sections were microwaved for 10 min for “antigen retrieval” and incubated with Ki67 antibody (1:200), followed by visualization with DAB using a Streptavidin-Horseradish Peroxidase kit. **Lower**, TUNEL labeling for apoptosis analysis. TUNEL labeling was performed with a fluorescent *in situ* cell death detection kit, according to the manufacturer’s instruction (Roche Applied Science, Germany).

Representative tumors harvested from each group were processed for histological analyses. Pathologic analysis (via H&E staining) found that there were large areas of necrosis in the tumor tissue treated with Ad-IFNγ, while few necrotic areas were observed in the Ad-LacZ-treated and PBS-treated tumors (data not shown). Cell proliferation was estimated by the immunohistochemical assessment of the nuclear antigen Ki-67. The results showed that Ki-67 was significantly lower in tumor tissue treated with Ad-IFNγ than those in Ad-LacZ group and PBS group. For apoptosis analysis, TUNEL positive ratios were significantly higher in Ad-IFNγ groups than those in Ad-LacZ group and PBS group. The representative pictures for Ki-67 and TUNEL staining in PBS group, Ad-LacZ group, and 1×10^9^ pfu Ad-IFNγ group were shown in Figure 6.

## Discussion

Interferon-γ (IFN-γ), as a multifunctional cytokine, exerts diverse biological functions related to host defense and immune regulation, such as inflammation, innate and acquired immunity, cell cycle and apoptosis [5]. IFN-γ plays a critical role in promoting protective host responses to tumors, which proposed mechanisms include, (a) anti-proliferative and pro-apoptotic actions, (b) anti-angiogenesis in tumors, and (c) promoting both the innate and adoptive immune responses against tumors [20,21].

Although IFN-γ has been investigated as a potential therapeutics for various types of tumors [6-13], the attempts to improve antitumor efficacy by increasing the dose or by repetitive continuous administration resulted in higher toxicity and low efficacy [22]. Considerable experimental data from our group and other investigators demonstrated that intratumoral IFN-γ gene transfer to achieve long-term, continuous locoregional exposure of IFN-γ is likely an appropriate approach for improving efficacy and reducing toxicity of IFN-γ [9,10,15,16,23,24]. In this study, we reported that adenovirus-mediated IFN-γ gene transfer (Ad-IFNγ) inhibited tumor growth of human nasopharyngeal carcinoma (NPC) cells (Figure 2 and Figure 5). Here the anti-NPC activity of IFN-γ included not only direct anti-proliferative and pro-apoptotic actions, also indirect mechanisms, such as immunomodulation and antiangiogenesis.

Ad-IFNγ efficiently expressed human IFN-γ in nasopharyngeal carcinoma cells *in vitro* and *in vivo* (Figure 1 and Figure 3), and exhibited strong antiproliferative effects (Figure 2A and Figure 6). According to previous reports, the antiproliferative mechanisms of IFN-γ seem to be cell type specific [9,25-29], either induction of cell cycle arrest or apoptosis. Here we found that both G1 phase arrest and apoptosis contributed to Ad-IFNγ-mediated growth suppression in NPC cell lines (Figure 2B, Figure 3 and Figure 6), consistent with the report that the antiproliferative effects of minicircle-IFNγ on NPC cell lines could be attributed to G1 arrest and apoptosis [9]. The JAK/STAT pathway may be responsible for most biological effects mediated by IFN-γ [20,30], which regulates different cell cycle-associated proteins that control the G1-S checkpoint [25], or induce cell apoptosis through up-regulating the expression of various apoptosis-related proteins in different cell types [31-33].

Although antiproliferation and apoptosis induction were main effects of Ad-IFNγ-mediated anti-NPC in this study, antiangiogenesis may contribute also to growth inhibition of NPC xenografts in nude mice. Because the microvessel densities (MVDs) were found to be decreased in the xenografts treated with Ad-IFNγ compared with those treated with Ad-LacZ or PBS in our study (data not shown). Previous studies reveal that IP-10, an inhibitor of angiogenesis, could be induced by IFN-γ in endothelial cells and exert potent antiangiogenesis activity by inhibiting endothelial cell differentiation, motility and tube formation [34,35]. However, due to highly species specificity, human unlikely exerts direct effects on murine vascular system, but likely acts on tumor cells to indirectly regulate angiogenesis. It was reported that IFN-γ acted as antiangiogenic cytokine by inhibiting the expression of angiogenic factors, such as VEGF or perlecan, in renal cell carcinoma [36], stromal fibroblasts [37], human cornea [38], and WiDr/HT29 colon carcinoma cells[39], or by inducing the expression of anti-angiogenic factor monokine induced by interferon-gamma (MIG, or CXCL9) in non-small cell lung carcinom [40]. Here we would like to make a hypothesis that IFN-γ may exert antiangiogenic effect in nude mice carrying NPC xenografts by regulating the expression of some angiogenic or antiangiogenic factors in NPC cells. This hypothesis, of course, needs to be tested and verified.

Human and murine IFN-γ display low level of sequence homology (only 40%) at protein level, which explains why the human and murine proteins display a strict species specificity in their ability to bind to and activate human and murine cells [41]. So human IFN-γ has less activity in the nude mouse host, though nude mice display potent macrophage and NK cell activity [42-44] and remain some basal T-cell function [45]. Direct immunomodulation on mouse immune system by human IFN-γ unlikely contributes to its antitumor effects. However, we cannot exclude the possibility that other indirect effects are involved in Ad-IFNγ-mediated antitumor immune response. Firstly, human IFN-γ could modify the expression of MHC and costimulatory molecules or cytokines, chemokines on human NPC cells [21,46], which may activate the residual immune system of nude mice. Secondly, adenoviral vector and heterogenous IFN-γ expression likely induce a nonspecific immune response directed by NK and/or microphage cells in nude mice [47].

Taken together, Ad-IFNγ displayed efficient anti-NPC activities by inhibiting tumor cell proliferation and induced cell apoptosis in this study. Additional indirect effects on antiangiogenesis and immunomodulatory may also be involved in this antitumor activity. So IFN-γ gene therapy by a replication defective adenovirus encoding the human IFN-γ (Ad-IFNγ) is likely a potential novel therapeutics on comprehensive therapy of nasopharyngeal carcinoma.

Nevertheless, recent reports have shown that the immune response activated by IFN-γ plays a dual role in cancer: It can not only suppress tumor growth by destroying cancer cells or inhibiting their outgrowth but also promote tumor progression either by selecting for tumor cells that are more fit to survive in an immunocompetent host or by establishing conditions within the tumor microenvironment that facilitate tumor outgrowth [48]. IFN-γ treatment is a double-edged sword whose anti- and protumorigenic activities are dependent on the cellular, microenvironmental, and/or molecular context [30]. Thereby, more investigations should be carried out to clarify the influences of cellular, microenvironmental, immunological and molecular events on the anti-NPC effects of Ad-IFNγ before its clinical application.

## Conclusions

The results of the present study indicate that Ad-IFNγ likely performs potent anti-nasopharyngeal carcinoma effects by inhibiting cell proliferation, inducing G1 phase arrest and cell apoptosis. These findings have implications for the use of IFN-γ gene therapy mediated by replication defective adenoviral vector as a promising approach in the treatment of nasopharyngeal carcinoma.

## Abbreviations

IFN-γ: Interferon-gamma; rhIFNγ: Recombinant human interferon-gamma; Ad-IFNγ: A replication defective adenovirus encoding the human IFN-γ; Ad-LacZ: A replication defective adenovirus encoding β-galactosidase; NPC: Nasopharyngeal carcinoma; RT-PCR: Reverse transcription-polymerase chain reaction; ELISA: Enzyme-linked immunosorbent assay; MTT: (3-(4,5-Dimethylthiazol-2-yl)-2,5-diphenyltetrazolium bromide; PI: Propidium iodide; DMEM: Dulbecco’s Modified Eagle Medium; PBS: Phosphate buffered saline; FBS: Fetal bovine serum; H&E: Hematoxylin and eosin; MOIs: Multiplicities of infection; TUNEL: TdT-mediated dUTP-biotin nick end labeling; MVD: Microvessel density; MIG: Monokine induced by interferon-gamma; NK cell: Natural killer cell.

## Competing interests

The authors declare that they have no competing interests.

## Authors’ contributions

RL carried out the cell proliferation and apoptosis analysis, collected and analyzed all of results, and designed and drafted the article. YZ LcZ and HL performed the animal experiments. LZ evaluated the expression of IFN-γ by RT-PCR and ELISA. PZ carried out immunohistochemical assay. HL and LK made animal model. HH performed and validated the statistical data. JW and WH conceived and coordinated the work, and helped in drafting the manuscript. All authors have read and approved the final manuscript.

## References

[B1] Adham M Kurniawan AN Muhtadi AI Roezin A Hermani B Gondhowiardjo S Tan IB Middeldorp JM Nasopharyngeal carcinoma in Indonesia: epidemiology, incidence, signs, and symptoms at presentation Chin J Cancer 2012 31 185 196 22313595 10.5732/cjc.011.10328 PMC3777476

[B2] Xiao WW Han F Lu TX Chen CY Huang Y Zhao C Treatment outcomes after radiotherapy alone for patients with early-stage nasopharyngeal carcinoma Int J Radiat Oncol Biol Phys 2009 74 1070 1076 10.1016/j.ijrobp.2008.09.008 19231110

[B3] Pan XB Zhu XD Role of chemotherapy in stage IIb nasopharyngeal carcinoma Chin J Cancer 2012 31 573 578 22776232 10.5732/cjc.011.10433 PMC3777455

[B4] Boehm U Klamp T Groot M Howard JC Cellular responses to interferon-gamma Annu Rev Immunol 1997 15 749 795 10.1146/annurev.immunol.15.1.749 9143706

[B5] Schroder K Hertzog PJ Ravasi T Hume DA Interferon-gamma: an overview of signals, mechanisms and functions J Leukoc Biol 2004 75 163 189 14525967 10.1189/jlb.0603252

[B6] Kominsky SL Hobeika AC Lake FA Torres BA Johnson HM Down-regulation of neu/HER-2 by interferon-gamma in prostate cancer cells Cancer Res 2000 60 3904 3908 10919667

[B7] Sasagawa T Hlaing M Akaike T Synergistic induction of apoptosis in murine hepatoma Hepa1-6 cells by IFN-gamma and TNF-alpha Biochem Biophys Res Commun 2000 272 674 680 10.1006/bbrc.2000.2835 10860813

[B8] Detjen KM Farwig K Welzel M Wiedenmann B Rosewicz S Interferon gamma inhibits growth of human pancreatic carcinoma cells via caspase-1 dependent induction of apoptosis Gut 2001 49 251 262 10.1136/gut.49.2.251 11454803 PMC1728385

[B9] Wu J Xiao X Zhao P Xue G Zhu Y Zhu X Zheng L Zeng Y Huang W Minicircle-IFNgamma induces antiproliferative and antitumoral effects in human nasopharyngeal carcinoma Clin Cancer Res 2006 12 4702 4713 10.1158/1078-0432.CCR-06-0520 16899621

[B10] Zhao P Zhu YH Wu JX Liu RY Zhu XY Xiao X Li HL Huang BJ Xie FJ Chen JM Adenovirus-mediated delivery of human IFNgamma gene inhibits prostate cancer growth Life Sci 2007 81 695 701 10.1016/j.lfs.2007.05.028 17714738

[B11] Kane A Yang I Interferon-gamma in brain tumor immunotherapy Neurosurg Clin N Am 2010 21 77 86 10.1016/j.nec.2009.08.011 19944968

[B12] Windbichler GH Hausmaninger H Stummvoll W Graf AH Kainz C Lahodny J Denison U Muller-Holzner E Marth C Interferon-gamma in the first-line therapy of ovarian cancer: a randomized phase III trial Br J Cancer 2000 82 1138 1144 10735496 10.1054/bjoc.1999.1053 PMC2363351

[B13] Hastie C Interferon gamma, a possible therapeutic approach for late-stage prostate cancer? Anticancer Res 2008 28 2843 2849 19031923

[B14] Miller CH Maher SG Young HA Clinical use of Interferon-gamma Ann N Y Acad Sci 2009 1182 69 79 10.1111/j.1749-6632.2009.05069.x 20074276 PMC6574079

[B15] Dummer R Eichmuller S Gellrich S Assaf C Dreno B Schiller M Dereure O Baudard M Bagot M Khammari A Phase II clinical trial of intratumoral application of TG1042 (adenovirus-interferon-gamma) in patients with advanced cutaneous T-cell lymphomas and multilesional cutaneous B-cell lymphomas Mol Ther 2010 18 1244 1247 10.1038/mt.2010.52 20372104 PMC2889748

[B16] Wu J Xiao X Jia H Chen J Zhu Y Zhao P Lin H Huang W Dynamic distribution and expression in vivo of the human interferon gamma gene delivered by adenoviral vector BMC Cancer 2009 9 55 10.1186/1471-2407-9-55 19216804 PMC2667533

[B17] Liu RY Dong Z Liu J Yin JY Zhou L Wu X Yang Y Mo W Huang W Khoo SK Role of eIF3a in regulating cisplatin sensitivity and in translational control of nucleotide excision repair of nasopharyngeal carcinoma Oncogene 2011 30 4814 4823 10.1038/onc.2011.189 21625209 PMC3165083

[B18] O’Reilly MS Boehm T Shing Y Fukai N Vasios G Lane WS Flynn E Birkhead JR Olsen BR Folkman J Endostatin: an endogenous inhibitor of angiogenesis and tumor growth Cell 1997 88 277 285 10.1016/S0092-8674(00)81848-6 9008168

[B19] Li L Liu RY Huang JL Liu QC Li Y Wu PH Zeng YX Huang W Adenovirus-mediated intra-tumoral delivery of the human endostatin gene inhibits tumor growth in nasopharyngeal carcinoma Int J Cancer 2006 118 2064 2071 10.1002/ijc.21585 16287067

[B20] Ikeda H Old LJ Schreiber RD The roles of IFN gamma in protection against tumor development and cancer immunoediting Cytokine Growth Factor Rev 2002 13 95 109 10.1016/S1359-6101(01)00038-7 11900986

[B21] Dunn GP Ikeda H Bruce AT Koebel C Uppaluri R Bui J Chan R Diamond M White JM Sheehan KC Schreiber RD Interferon-gamma and cancer immunoediting Immunol Res 2005 32 231 245 10.1385/IR:32:1-3:231 16106075

[B22] Curnis F Gasparri A Sacchi A Cattaneo A Magni F Corti A Targeted delivery of IFNgamma to tumor vessels uncouples antitumor from counterregulatory mechanisms Cancer Res 2005 65 2906 2913 10.1158/0008-5472.CAN-04-4282 15805293

[B23] Gattacceca F Pilatte Y Billard C Monnet I Moritz S Le Carrou J Eloit M Jaurand MC Ad-IFN gamma induces antiproliferative and antitumoral responses in malignant mesothelioma Clin Cancer Res 2002 8 3298 3304 12374702

[B24] Dummer R Hassel JC Fellenberg F Eichmuller S Maier T Slos P Acres B Bleuzen P Bataille V Squiban P Adenovirus-mediated intralesional interferon-gamma gene transfer induces tumor regressions in cutaneous lymphomas Blood 2004 104 1631 1638 10.1182/blood-2004-01-0360 15161670

[B25] Sangfelt O Erickson S Grander D Mechanisms of interferon-induced cell cycle arrest Front Biosci 2000 5 D479 D487 10.2741/Sangfelt 10762599

[B26] Chawla-Sarkar M Lindner DJ Liu YF Williams BR Sen GC Silverman RH Borden EC Apoptosis and interferons: role of interferon-stimulated genes as mediators of apoptosis Apoptosis 2003 8 237 249 10.1023/A:1023668705040 12766484

[B27] Gollob JA Sciambi CJ Huang Z Dressman HK Gene expression changes and signaling events associated with the direct antimelanoma effect of IFN-gamma Cancer Res 2005 65 8869 8877 10.1158/0008-5472.CAN-05-1387 16204058

[B28] Wall L Burke F Barton C Smyth J Balkwill F IFN-gamma induces apoptosis in ovarian cancer cells in vivo and in vitro Clin Cancer Res 2003 9 2487 2496 12855622

[B29] Dunn GP Sheehan KC Old LJ Schreiber RD IFN unresponsiveness in LNCaP cells due to the lack of JAK1 gene expression Cancer Res 2005 65 3447 3453 15833880 10.1158/0008-5472.CAN-04-4316

[B30] Zaidi MR Merlino G The two faces of interferon-gamma in cancer Clin Cancer Res 2011 17 6118 6124 10.1158/1078-0432.CCR-11-0482 21705455 PMC3186825

[B31] Ruiz-Ruiz C de Almodovar Ruiz C Rodriguez A Ortiz-Ferron G Redondo JM Lopez-Rivas A The up-regulation of human caspase-8 by interferon-gamma in breast tumor cells requires the induction and action of the transcription factor interferon regulatory factor-1 J Biol Chem 2004 279 19712 19720 10.1074/jbc.M313023200 14993214

[B32] Miura Y Tsujioka T Nishimura Y Sakaguchi H Maeda M Hayashi H Dong M Hyodoh F Yata K Wada H TRAIL expression up-regulated by interferon-gamma via phosphorylation of STAT1 induces myeloma cell death Anticancer Res 2006 26 4115 4124 17201122

[B33] Barton C Davies D Balkwill F Burke F Involvement of both intrinsic and extrinsic pathways in IFN-gamma-induced apoptosis that are enhanced with cisplatin Eur J Cancer 2005 41 1474 1486 10.1016/j.ejca.2005.03.022 15949937

[B34] Bodnar RJ Yates CC Rodgers ME Du X Wells A IP-10 induces dissociation of newly formed blood vessels J Cell Sci 2009 122 2064 2077 10.1242/jcs.048793 19470579 PMC2723158

[B35] Bodnar RJ Yates CC Wells A IP-10 blocks vascular endothelial growth factor-induced endothelial cell motility and tube formation via inhibition of calpain Circ Res 2006 98 617 625 10.1161/01.RES.0000209968.66606.10 16484616 PMC3826264

[B36] Sasamura H Takahashi A Miyao N Yanase M Masumori N Kitamura H Itoh N Tsukamoto T Inhibitory effect on expression of angiogenic factors by antiangiogenic agents in renal cell carcinoma Br J Cancer 2002 86 768 773 10.1038/sj.bjc.6600152 11875741 PMC2375312

[B37] Lu Y Yang W Qin C Zhang L Deng J Liu S Qin Z Responsiveness of stromal fibroblasts to IFN-gamma blocks tumor growth via angiostasis J Immunol 2009 183 6413 6421 10.4049/jimmunol.0901073 19841170

[B38] Kommineni VK Nagineni CN William A Detrick B Hooks JJ IFN-gamma acts as anti-angiogenic cytokine in the human cornea by regulating the expression of VEGF-A and sVEGF-R1 Biochem Biophys Res Commun 2008 374 479 484 10.1016/j.bbrc.2008.07.042 18639520 PMC2997485

[B39] Sharma B Iozzo RV Transcriptional silencing of perlecan gene expression by interferon-gamma J Biol Chem 1998 273 4642 4646 10.1074/jbc.273.8.4642 9468523

[B40] Addison CL Arenberg DA Morris SB Xue YY Burdick MD Mulligan MS Iannettoni MD Strieter RM The CXC chemokine, monokine induced by interferon-gamma, inhibits non-small cell lung carcinoma tumor growth and metastasis Hum Gene Ther 2000 11 247 261 10.1089/10430340050015996 10680839

[B41] Farrar MA Schreiber RD The molecular cell biology of interferon-gamma and its receptor Annu Rev Immunol 1993 11 571 611 10.1146/annurev.iy.11.040193.003035 8476573

[B42] Zhang F Lu W Dong Z Tumor-infiltrating macrophages are involved in suppressing growth and metastasis of human prostate cancer cells by INF-beta gene therapy in nude mice Clin Cancer Res 2002 8 2942 2951 12231540

[B43] Nielsen LL NK cells mediate the anti-tumor effects of E1-deleted, type 5 adenovirus in a human tumor xenograft model Oncol Rep 2000 7 151 155 10601610

[B44] Sarkar D Su ZZ Vozhilla N Park ES Randolph A Valerie K Fisher PB Targeted virus replication plus immunotherapy eradicates primary and distant pancreatic tumors in nude mice Cancer Res 2005 65 9056 9063 10.1158/0008-5472.CAN-05-1261 16204080

[B45] Ikehara S Pahwa RN Fernandes G Hansen CT Good RA Functional T cells in athymic nude mice Proc Natl Acad Sci U S A 1984 81 886 888 10.1073/pnas.81.3.886 6608104 PMC344943

[B46] Seliger B Ruiz-Cabello F Garrido F IFN inducibility of major histocompatibility antigens in tumors Adv Cancer Res 2008 101 249 276 19055946 10.1016/S0065-230X(08)00407-7 PMC7125809

[B47] Hu X Chakravarty SD Ivashkiv LB Regulation of interferon and Toll-like receptor signaling during macrophage activation by opposing feedforward and feedback inhibition mechanisms Immunol Rev 2008 226 41 56 10.1111/j.1600-065X.2008.00707.x 19161415 PMC2630590

[B48] Schreiber RD Old LJ Smyth MJ Cancer immunoediting: integrating immunity’s roles in cancer suppression and promotion Science 2011 331 1565 1570 10.1126/science.1203486 21436444

